# Acinetobacter Metabolism in Infection and Antimicrobial Resistance

**DOI:** 10.1128/iai.00433-22

**Published:** 2023-05-16

**Authors:** Xiaomei Ren, Lauren D. Palmer

**Affiliations:** a Department of Microbiology and Immunology, University of Illinois Chicago, Chicago, Illinois, USA; University of California at Santa Cruz, Department of Microbiology and Environmental Toxicology

**Keywords:** *Acinetobacter*, metabolism, nutrients, infection, antimicrobial, *A. baumannii*, antibiotic resistance, antimicrobial resistance

## Abstract

Acinetobacter infections have high rates of mortality due to an increasing incidence of infections by multidrug-resistant (MDR) and extensively-drug-resistant (XDR) strains. Therefore, new therapeutic strategies for the treatment of Acinetobacter infections are urgently needed. Acinetobacter spp. are Gram-negative coccobacilli that are obligate aerobes and can utilize a wide variety of carbon sources. Acinetobacter baumannii is the main cause of Acinetobacter infections, and recent work has identified multiple strategies A. baumannii uses to acquire nutrients and replicate in the face of host nutrient restriction. Some host nutrient sources also serve antimicrobial and immunomodulatory functions. Hence, understanding Acinetobacter metabolism during infection may provide new insights into novel infection control measures. In this review, we focus on the role of metabolism during infection and in resistance to antibiotics and other antimicrobial agents and discuss the possibility that metabolism may be exploited to identify novel targets to treat Acinetobacter infections.

## INTRODUCTION

Acinetobacter spp. are a major cause of opportunistic infections. Acinetobacter spp. are Gram-negative coccobacilli that are nonfermentative, oxidase negative, indole negative, catalase positive, and lack flagella but are capable of twitching motility (1). Acinetobacter spp. are *Gammaproteobacteria* in the order *Pseudomonadales* and family *Moraxellaceae.* Acinetobacter baumannii is the primary pathogen among Acinetobacter spp. and is a threat to public health due to widespread incidence of multidrug-resistant (MDR) and increasing extensively-drug-resistant (XDR) strains. A. baumannii can infect any site in the body and is a frequent cause of urinary tract infections, bloodstream infections, and ventilator-associated pneumonia in critically ill patients, contributing to increased morbidity and mortality (2–4). In the United States, A. baumannii cases decreased in 2018 and 2019, but they have recently risen following the COVID-19 pandemic (5). A. baumannii can also cause community-acquired infections, and while these isolates are less likely to be MDR, the infections can be severe (6, 7). Acinetobacter spp. are often found in the environment, but the reservoir of A. baumannii and closely related pathogenic strains appears to be human associated (2). A. baumannii colonization of the gut, nasopharynx, skin, and upper respiratory tract is associated with increased risk for invasive infection and vice versa (8–16). Additionally, A. baumannii is adapted to withstand the hospital environment, with exceptional resistance to disinfectants and desiccation, which also promote virulence (17–19).

The MDR rates for A. baumannii infection isolates range from 47% to 93% according to a 2016 report (20). Clinically, A. baumannii has broad-spectrum resistance to a range of β-lactams, aminoglycosides, fluoroquinolones, and even the last resort antimicrobial colistin (21, 22). Due to the wide spread of MDR A. baumannii, few antibiotics are effective for treating infections caused by this pathogen (23). In 2017, A. baumannii was included on the list of “Priority Pathogens” by the World Health Organization (WHO) for development of novel antibiotics (24). The U.S. Centers for Disease Control and Prevention maintains carbapenem-resistant A. baumannii as an urgent threat (25). Therefore, development of new strategies to treat A. baumannii infections is a critical challenge.

During infection and colonization, the host serves as the sole source of nutrition to invading pathogens. Host metabolism and bacterial metabolism therefore represent potential therapeutic targets to limit bacterial replication and support host immunity. Many early studies investigated the physiology and metabolism of Acinetobacter strains and were previously reviewed by Juni in 1978 (26). While many of the general features of Acinetobacter metabolism were established in the 1960s and 1970s, recent work has uncovered the role of Acinetobacter metabolism in infection and antibiotic resistance. Genome-scale mutant analyses such as transposon sequencing (Tn-seq) and insertion sequencing (INseq) have shown that metabolic genes are essential during infection. Nutrient acquisition and metabolism were major contributors to A. baumannii infection and persistence in a murine model of lung infection and a Galleria mellonella model of infection (27, 28). Nutrient iron acquisition was also identified through Tn-seq to contribute to the fitness of A. baumannii during bloodstream infection (29). In this review, we will provide an overview of unique features of Acinetobacter metabolism and discuss work that has identified critical nutrient sources during infection and how central metabolism interacts with antibiotic resistance.

## GENERAL FEATURES OF ACINETOBACTER METABOLISM

Acinetobacter spp. are nonfermentative and strictly aerobic (26). However, A. baumannii has been reported to survive at least 28 days in the absence of oxygen, suggesting mechanisms for persistence in anaerobic environments (30). Acinetobacter calcoaceticus (then named Micrococcus calcoaceticus) was first described in 1911 and was isolated in minimal medium with either acetate or quinate as the carbon source (31, 32). Early studies noted that vigorous aeration, lower pH (5.5), and nitrate as a nitrogen source promoted enrichment of Acinetobacter spp. (32). In addition to nitrate, Acinetobacter spp. can use ammonium and nitrite as inorganic nitrogen sources (33). The vast majority of Acinetobacter isolates are prototrophs that are capable of growing in minimal medium and do not require exogenous vitamins, amino acids, or nucleotides (32–34). Acinetobacter spp. are typically capable of growing at 37°C, and some strains grow up to 44°C, while some environmental strains grow only up to 30°C (1, 35). Acinetobacter strains can assimilate a broad range of carbon sources, including sugars, organic acids, amino acids, and ethanol (36–39). While each Acinetobacter strain uses a broad range of carbon sources, there is considerable variability in the specific carbon sources that can be assimilated.

**FIG 1:**
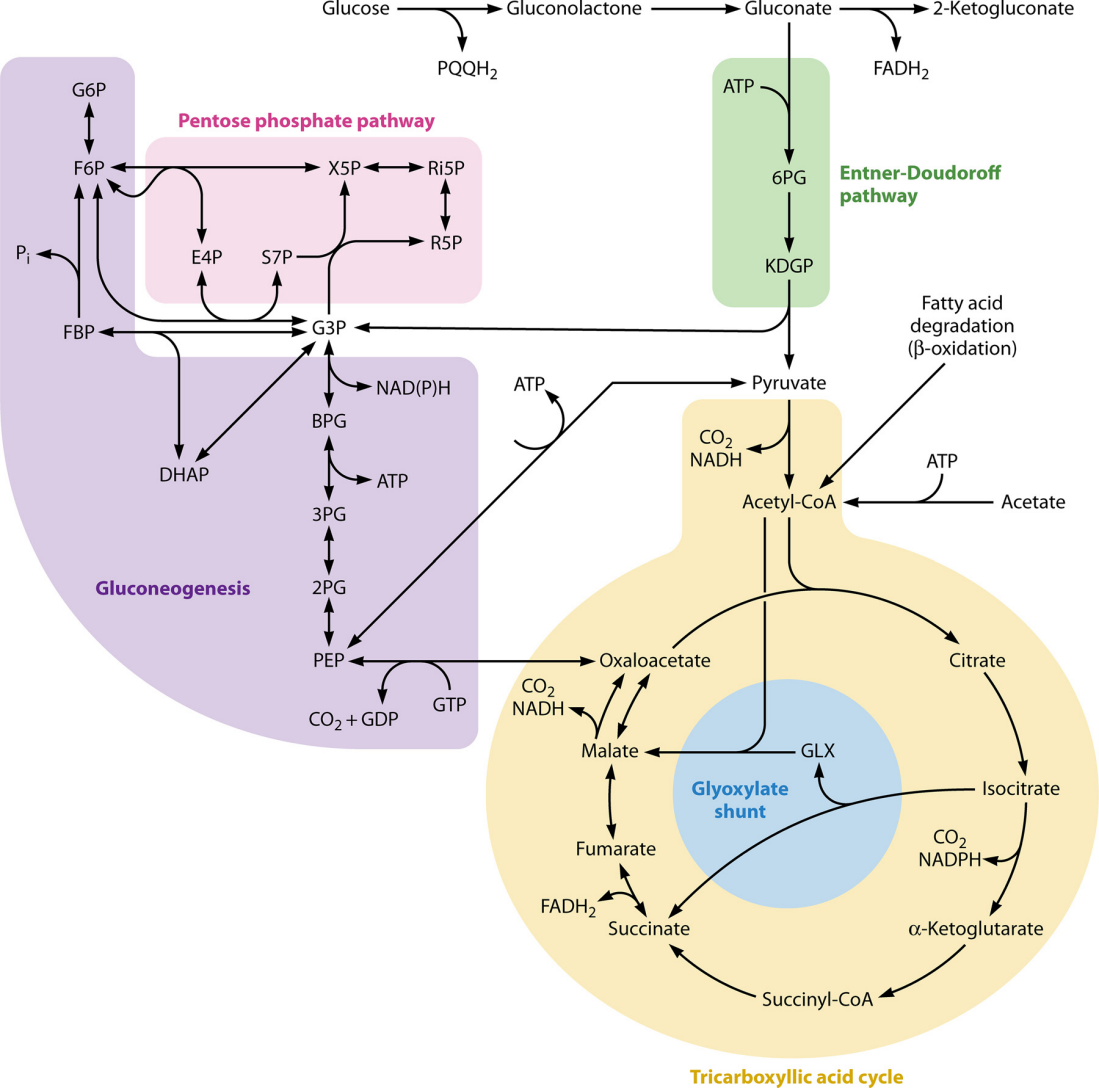
Central carbon metabolism in pathogenic Acinetobacter. The gray arrow indicates that gluconolactone can be nonenzymatically hydrolyzed to gluconate and the enzyme is not typically encoded in A. baumannii and closely related species. Abbreviations: PQQH2, reduced pyrroloquinoline quinone; 6PG, 6-phosphogluconate; KDGP, 2-keto-3-deoxy-6-phosphogluconate; G6P, glucose-6-phosphate; F6P, fructose-6-phosphate; FBP, fructose-1,6-bisphosphate; E4P, erythrose-4-phosphate; S7P, sedulose-7-phosphate; X5P, xylose-5-phosphate; Ri5P, ribulose-5-phosphate; R5P, ribose-5-phosphate; G3P, glyceraldehyde-3-phosphate; DHAP, dihydroxyacetone phosphate; BPG, 1,3-bisphosphoglycerate; 3PG, 3-phosphoglycerate; 2PG, 2-phosphoglycerate; PEP, phosphoenolpyruvate; GLX, glyoxylate.

Acinetobacter spp. use the Entner-Doudoroff pathway, the pentose phosphate pathway, the tricarboxylic acid (TCA) cycle, the glyoxylate pathway, and gluconeogenesis in central carbon metabolism (reviewed in reference 26) (Fig. 1). Notably, most Acinetobacter spp. are not capable of utilizing glucose as a carbon source, as first reported by Baumann et al. in 1968, and do not appear to encode hexose kinases (33, 36, 37, 40–42). Indeed, when the type strain, A. baumannii ATCC 17978, was sequenced, the authors discovered this strain lacked hexokinase, glucokinase, or a glucose-specific phosphotransferase system (PTS) (43). Acinetobacter spp. that can utilize glucose as a carbon source use the following pathway: (i) glucose is oxidized by a nonspecific pyrroloquinoline quinone (PQQ)-dependent aldose dehydrogenase that produces gluconolactone, (ii) gluconolactone is enzymatically or nonenzymatically hydrolyzed to gluconate, and (iii) gluconate is phosphorylated and enters the Entner-Doudoroff pathway (42, 44). A small number of A. baumannii strains, including A. baumannii ATCC 17978, do not encode the PQQ biosynthesis cluster and likely lost the glucose dehydrogenase activity (45). However, many A. baumannii strains that cannot utilize glucose as a carbon and energy source are still able to degrade glucose to gluconolactone/gluconate; this reaction can be readily detected by the acidification of medium in the presence of d-glucose (1, 26, 46). These strains are unable to utilize glucose because they lack the ability to degrade gluconate (47). The role of glucose oxidation without assimilation in the physiology of pathogenic Acinetobacter spp. is unknown.

Acinetobacter spp. also notably degrade many organic compounds by dissimilatory pathways. Environmental strains such as Acinetobacter baylyi ADP1 are well studied for their ability to oxidize aromatic compounds by the β-ketoadipate pathway and potential for bioengineering (reviewed previously in references 26, 48, and 49). One study found that catabolism of citric acid, serine, and glucose correlated with phylogeny, suggesting diversification of Acinetobacter carbon assimilation has played a crucial role in evolution of the genus (50). How pathogenic Acinetobacter spp. regulate carbon source prioritization has not been well studied. In the model organisms Escherichia coli and Bacillus subtilis, carbon catabolite repression is regulated by the catabolite repressor protein (Crp), cAMP, and enzyme IIA of the glucose-specific PTS system (51, 52). Acinetobacter spp. encode a catabolite repressor protein (Crp) homolog that is a major target of persulfidation in the presence of exogenous sulfide stress (53). A. baumannii Crp is uncharacterized. Acinetobacter spp. do not encode the glucose-specific PTS system, as noted above; therefore, Acinetobacter carbon catabolite repression mechanisms are likely distinct from those of E. coli and B. subtilis. The environmental strain A. baylyi encodes a catabolite repression control (Crc) protein that represses aromatic compound degradation in the presence of organic acids such as succinate and acetate by multiple mechanisms, including transcription-independent mechanisms (54–56). Pseudomonas spp. are in the same order as the Acinetobacter genus and also use succinate as a preferred carbon source; Crc is involved in carbon catabolite repression, while the Crp homolog, PTS system, and cAMP are not (reviewed in references 52 and 57). Therefore, some environmental Acinetobacter spp. prioritize organic acid catabolism, but the mechanisms of carbon catabolite repression and its role in infection are understudied in pathogenic Acinetobacter spp.

In summary, some features of Acinetobacter metabolism are generally conserved, including obligate aerobic metabolism, prototrophy, encoding central carbon metabolism pathways including Entner-Doudoroff, and utilization of diverse carbon and energy sources. The specific sets of compounds each species and strain can utilize for carbon, energy, nitrogen, and other nutrient sources vary considerably, and nutrient utilization likely confers specific niche advantages in Acinetobacter infection and pathogenesis.

## METALS AS HOST-RESTRICTED MICRONUTRIENTS AND INTOXICANTS

**FIG 2:**
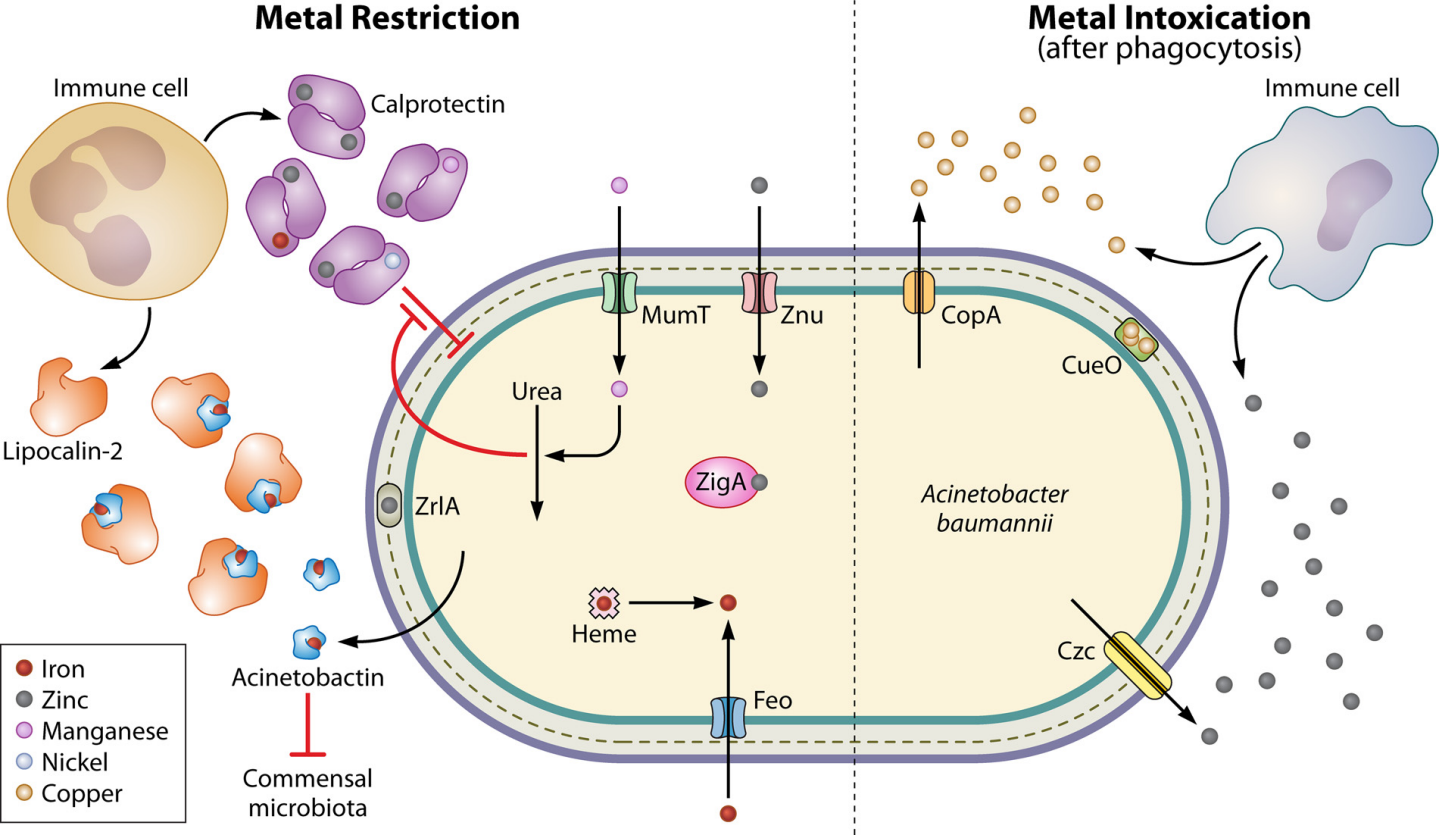
Host-mediated nutrient metal restriction and intoxication during A. baumannii infection. (Left side) Metal restriction. Host innate immune cells such as neutrophils release calprotectin, which binds zinc, manganese, iron, and nickel, limiting pathogen access. A. baumannii imports manganese and zinc with MumT and Znu transporters. Urea metabolism is coordinated with the response to manganese restriction and helps resist calprotectin. A. baumannii relies on the siderophore acinetobactin to acquire iron during infection, and in some sites, acinetobactin iron acquisition is disrupted by host release of lipocalin-2. Acinetobactin also helps A. baumannii competitively inhibit growth of commensal microbiota members. A. baumannii can also utilize heme and ferrous iron transport system Feo to acquire iron. (Right side) Metal intoxication. The host also imposes metal intoxication on invading A. baumannii, likely after phagocytosis. A. baumannii fights copper intoxication with the CopA efflux protein and CueO multicopper oxidase. Excess zinc depletes A. baumannii copper, and A. baumannii resists zinc intoxication with the Czc efflux proteins.

Invading pathogens must acquire nutrient metals from the host, and the host has evolved mechanisms to prevent bacterial metal acquisition, termed “nutritional immunity” (58–60). Acquisition of nutrient metals, including iron, manganese, and zinc, has been identified as a critical mediator of A. baumannii pathogenesis and has been discussed in previous reviews (61, 62). We will therefore briefly highlight recent studies. Multimetal binding by the innate immune protein calprotectin contributes to nutritional immunity in the host (Fig. 2). Calprotectin-mediated zinc sequestration activates a complex programmed transcriptional response primarily mediated by the zinc uptake regulator (Zur), which includes activation of genes encoding the zinc uptake (Znu) transporters, the putative zinc metallochaperone ZigA, and the peptidase ZrlA, which maintains cell wall integrity; Zur, ZnuABCD, ZigA, and ZrlA are all required for infection in a murine model of pneumonia (63–68). These zinc-responsive systems are interconnected with other areas of metabolism. For example, ZigA is also important for utilization of histidine as a carbon source in zinc-deplete medium (67); ZrlA is also important for outer membrane vesicle production, epithelial cell adherence, and resistance to membrane stresses and antibiotics, including meropenem administered during mouse lung infection (65, 66, 69). Additionally, zinc restriction compromises A. baumannii flavin biosynthesis, emphasizing the integration of metal and central metabolism (70). Calprotectin also limits pathogen access to manganese, which A. baumannii combats with the manganese and urea metabolism (Mum) system. The manganese import protein MumT is required for lung infection and dissemination to the liver in mice; however, MumT is not required in calprotectin-deficient mice (71). *mumT* is activated by MumR, a manganese-responsive transcriptional regulator required for oxidative stress resistance, urea catabolism (e.g., the urea carboxylase MumC), and murine lung infection (71, 72). Together, these findings suggest that A. baumannii responds to host-mediated nutrient metal restriction by upregulating systems that allow carbon and nitrogen assimilation and promote resistance to host stresses and antibiotics.

A. baumannii encodes multiple iron acquisition systems. The ferrous iron acquisition system FeoABC has been demonstrated to be important for A. baumannii colonization and persistence in mouse bloodstream and pneumonia models of infection, but was not important for mortality during bloodstream infection (29, 73, 74). Some strains encode a heme acquisition system that also liberates iron from heme, likely contributing to virulence (75). In order to acquire ferric iron in the presence of host metal restriction, many bacteria use siderophores, which are small molecules that bind iron with high affinity (76). Biosynthesis of the mixed-type hydroxamate/catecholate siderophore acinetobactin has been shown to be essential for A. baumannii virulence in a mouse model of bloodstream infection and the G. mellonella infection model (77–79). Lipocalin-2 is an additional nutritional immunity protein that sequesters many bacterial siderophores (58–60). Lipocalin-2 can inhibit acinetobactin-dependent growth *in vitro*, and mice lacking lipocalin-2 have increased mortality in murine bloodstream infection and increased bacterial burdens in lung infection (80). Acinetobactin biosynthesis appears to be acquired in clinical A. baumannii isolates compared to environmental strains which have TonB-dependent xenosiderophore acquisition instead (81). Interestingly, A. baumannii can also use acinetobactin to inhibit growth of commensal skin and upper respiratory tract bacteria *in vitro*, uncovering a mechanism that may promote A. baumannii asymptomatic colonization (82). These studies show that A. baumannii siderophore biosynthesis is critical to its pathogenesis and may contribute to asymptomatic carriage and dissemination. Siderophore-mediated bacterial iron acquisition was targeted by the new antibiotic cefiderocol, a so-called “Trojan horse” catechol siderophore cephalosporin (83, 84). Cefiderocol is approved for treating Gram-negative infections, including complicated urinary tract infections by carbapenem-resistant bacteria (85). Unfortunately, cefiderocol was associated with higher all-cause mortality in A. baumannii infections in a phase 3 trial and 50% resistance in a retrospective trial, which may be explained in part by widespread heteroresistance—in which a subpopulation of the bacteria is resistant—in carbapenem-resistant A. baumannii (86–90). While cefiderocol may not be optimally effective for treatment of all A. baumannii infections, it remains a useful antibiotic for treating Gram-negative bacterial infections and emphasizes the utility of nutrient acquisition as an antimicrobial target.

In addition to restricting pathogen access to metals, innate immune cells can kill invading bacteria by intoxication of metal such as copper or zinc (91). A. baumannii encodes many copper resistance genes, including the copper efflux protein CopA and the multicopper oxidase CueO that are essential for virulence in a G. mellonella infection model (92). Consistent with these findings, CopA was required for colonization and persistence in a murine model of lung infection (93). Another study found that the CueO multicopper oxidase (sometimes annotated as CopA) was not required for macrophage infection by the strain A. baumannii 19606 (94); however, A. baumannii 19606 cannot replicate in macrophages (95). Whether copper oxidation contributes to survival in macrophages by strains that can replicate intracellularly has yet to be determined. Zinc efflux has also been shown to be important in A. baumannii infection. A mutant lacking the cobalt-zinc-cadmium export protein CzcA infected the respiratory tract with higher burdens than the wild type but was defective in dissemination in a mouse model of dietary zinc deficiency and lung infection (96). Interestingly, zinc intoxication has been linked with copper depletion, and copper supplementation can prevent killing of A. baumannii by THP-1 macrophage-like cells, suggesting zinc intoxication is a critical mediator of macrophage-mediated A. baumannii killing (97). Together, these recent findings highlight the important role of nutrient metals at the host-pathogen interface in A. baumannii infections.

## LIPIDS AS ANTIMICROBIALS AND NUTRIENT SOURCES

**FIG 3:**
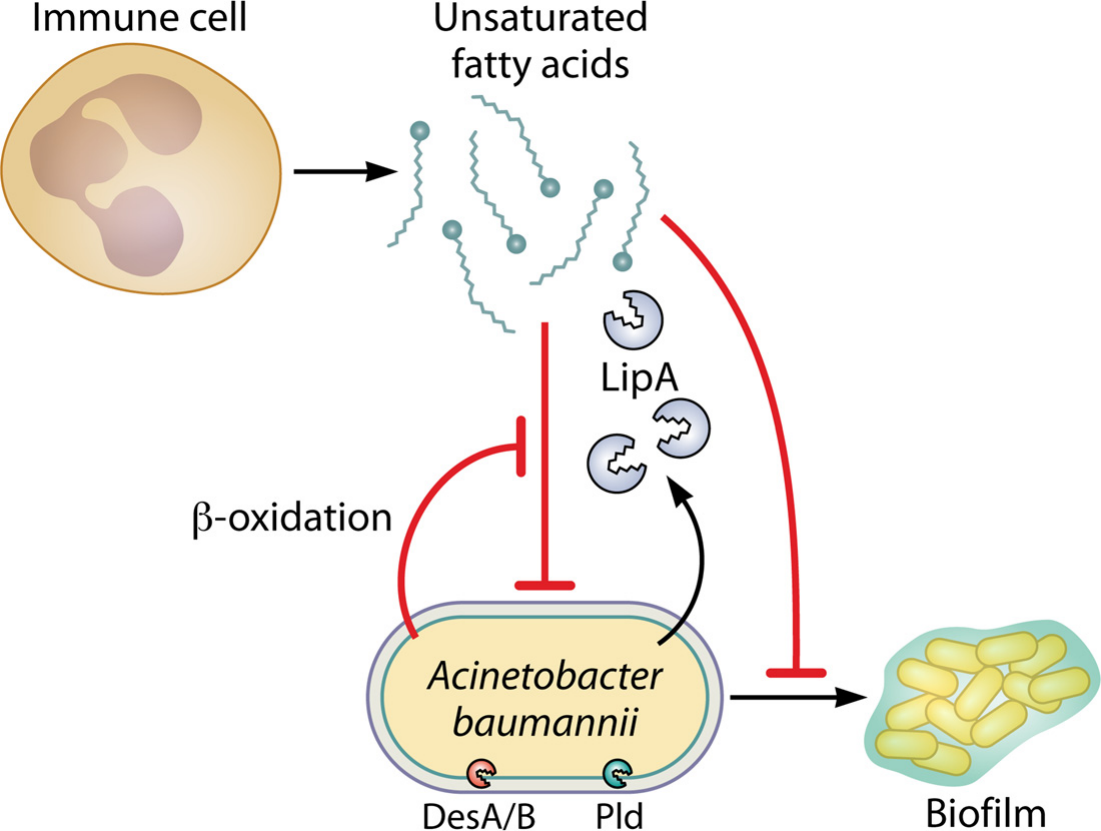
Long-chain fatty acids mediate opposing effects during A. baumannii infection. A. baumannii desaturases DesA/B and phospholipase D (Pld) are critical during infection. During inflammatory bursts immune cells such as neutrophils can release polyunsaturated fatty acids (PUFAs) that inhibit bacteria, including A. baumannii. A. baumannii β-oxidation can help combat PUFA toxicity. Host monounsaturated fatty acids can inhibit A. baumannii quorum sensing and biofilm formation.

All Acinetobacter spp. synthesize lipids, including isoprenoid precursors, lipid A, and long-chain fatty acids, as essential components of their inner and outer membranes. Phospholipase D activity, including synthesis of cardiolipin fatty acids, is essential for A. baumannii virulence in G. mellonella, mouse lung, and human epithelial cell infection models and for resistance to the last resort antibiotic colistin (Fig. 3) (98–100). A. baumannii encodes two desaturase enzymes that are important for maintaining membrane fluidity: in a murine model of lung infection, the desaturase DesA is critical for colonization and persistence in the lung, while desaturase DesB is critical for dissemination to other organs (101). A. baumannii maintains outer membrane integrity in part through the maintenance of the lipid asymmetry (Mla) system, which contributes to intrinsic antibiotic resistance (102–105). In a murine model of lung infection, isolation of a suppressor mutant in the Δ*mlaF* A. baumannii ATCC 17978UN strain uncovered synthetic interactions between isoprenoid biosynthesis and the Mla system in A. baumannii that promote virulence and resistance to antibiotics and host stresses (105, 106). Therefore, A. baumannii lipid synthesis and metabolism are important for virulence.

During infection, host fatty acids can serve as antimicrobial compounds inducing fatty acid stress or as nutrient sources. Host long-chain polyunsaturated fatty acids (PUFAs) such as arachidonic acid (AA) and decosahexaenoic acid (DHA) are released during inflammatory bursts of macrophages and neutrophils (107–109). PUFAs have antimicrobial properties that A. baumannii β-oxidation helps resist, suggesting A. baumannii may metabolize the toxic PUFAs (110). Monounsaturated fatty acids have also been shown to decrease A. baumannii quorum sensing and biofilm formation (111). In addition, recent studies found that supplementing PUFAs *in vitro* can slow down the rate at which A. baumannii acquires resistance to erythromycin and tetracycline, suggesting fatty acid supplements provide a novel way to limit the development of resistance in A. baumannii (112, 113).

By contrast, A. baumannii can use fatty acids directly in membrane biogenesis or as a carbon source or energy sources. Phospholipids are a major component of the Gram-negative bacterial cell envelope and are synthesized by phosphatidylglycerol phosphate (PGP) phosphatases. In *A. bauamnnii*, two PGP enzymes, PGPA and PGPB, have been identified (114). The *pgpA* mutant strains had altered distribution of phosphatidylethanolamine (PE) phospholipid species and increased susceptibility to gentamicin (114). The *pgpB* mutant strains were more susceptible to carbapenem antibiotics (114). These findings suggest that PGP enzymes play an important role in maintaining the structural and functional integrity of the bacterial cell envelope and that targeting these enzymes could potentially lead to effective combination antimicrobial therapies. A. baumannii relies largely upon fatty acid acquisition from the host in most organ niches except blood during infection (115). Choline and phosphatidylcholine can serve as energy sources (measured by increased ATP production), which is dependent on the betaine-choline-carnitine transporters (116). A. baumannii can also use fatty acids as carbon and energy sources with the lipase LipA (117). LipA is secreted by the type 2 secretion system (T2SS) and is essential for long-chain fatty acid utilization and colonization and persistence in a mouse bloodstream infection model (117). Similar to metals at the host-pathogen interface, fatty acids therefore have the potential to inhibit A. baumannii infection or be metabolized as energy and/or carbon sources.

## ORGANIC ACIDS AS CARBON SOURCES AND IMMUNOMODULATORS

Organic acids are used as carbon sources by many Acinetobacter strains. An early study surveying the metabolic capabilities found that only five compounds supported the growth of all 106 Acinetobacter strains tested: the organic acids/short-chain fatty acids acetate, butyrate, pentanoate (also known as valerate), hexanoate (also known as caproate), and pyruvate (33). Most strains of Acinetobacter can use many organic acids as carbon and energy sources, including *trans*-aconitate, adipate, azelate, benzoate, caprate, citrate, fumarate, glutarate, α-ketoglutarate, dl-lactate, malonate, succinate, and phenylacetate, among others (1, 33). For many of these organic acids, their role in virulence has not been explored. As mentioned above, the environmental species A. baylyi and Pseudomonas species prioritize organic acid catabolism by carbon catabolite repression (54–57). However, the role of carbon catabolite repression is uncharacterized in pathogenic Acinetobacter spp.

**FIG 4:**
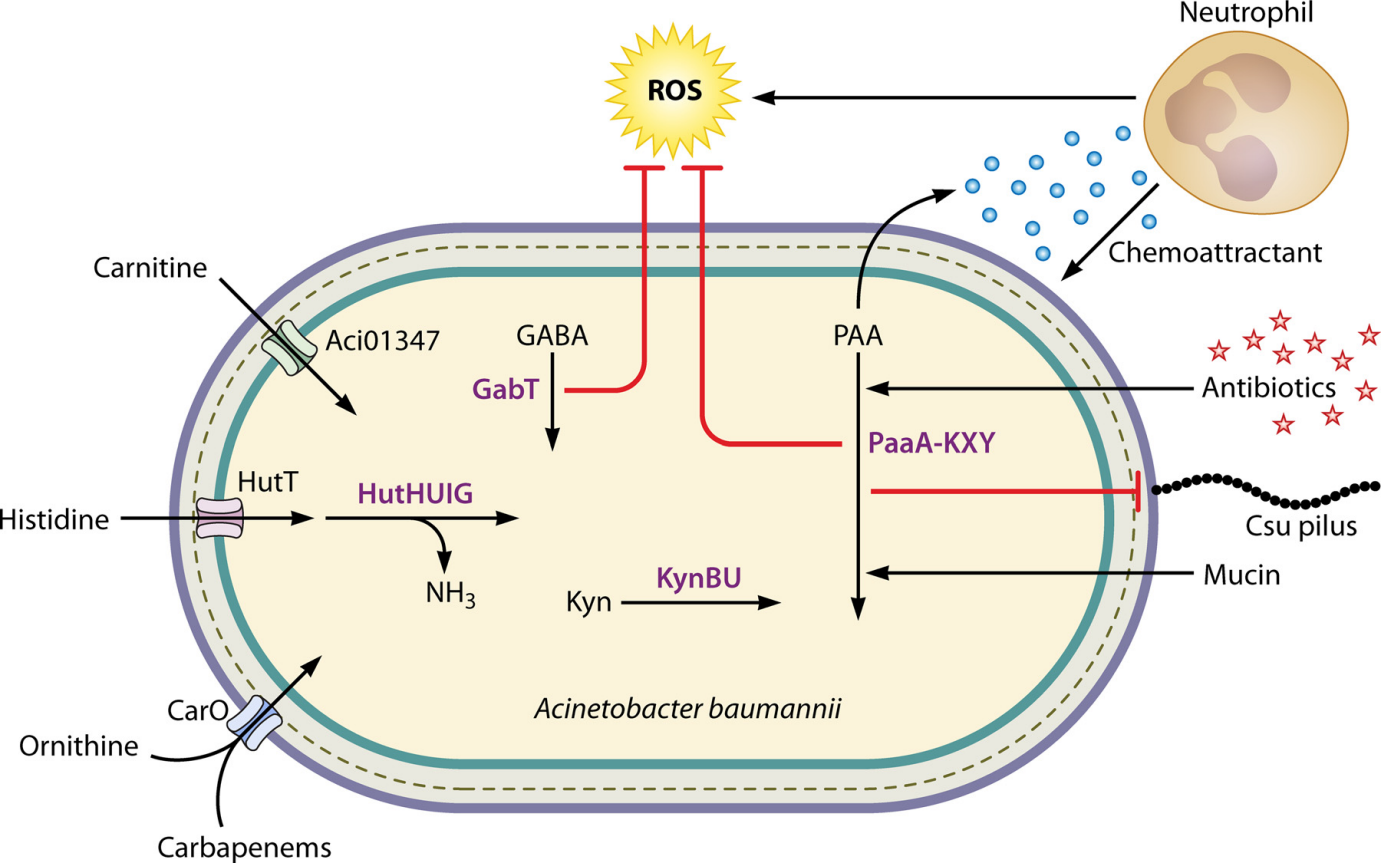
Catabolism of organic acids and amine compounds promotes A. baumannii virulence. A. baumannii encodes carnitine import protein Aci01347, which is required for growth on carnitine as the sole carbon and energy source. Histidine catabolism is encoded by pathogenic Acinetobacter spp., and HutH is required for utilization of histidine as a carbon and nitrogen source. Mutants lacking CarO are resistant to carbapenems and lose ornithine transport activity, suggesting CarO transports carbapenems and ornithine into the cell. Pathogenic Acinetobacter spp. can catabolize kynurenine (Kyn) with KynB and KynU. The γ-aminobutyric acid (GABA) aminotransferase GabT and the phenylacetic acid (PAA) pathway both contribute to resistance to reactive oxygen species (ROS). PAA catabolism also contributes to immune evasion, as mutants in the pathway excrete PAA, which serves as a neutrophil chemoattractant. Antibiotics promote expression of PAA pathway genes and inhibit chaperone-usher (Csu) pilus expression in a PAA-dependent mechanism. Host mucin glycoproteins can serve as the sole carbon and energy source for A. baumannii and promote *paa* gene expression.

Organic acid catabolism has been shown to be essential for A. baumannii virulence and immune modulation (Fig. 4). Pyruvate catabolism in the presence of human pleural fluid increased A. baumannii cytotoxicity and killing of human epithelial cells and murine macrophages (118). Acinetobacter catabolism of the organic acid phenylacetate (phenylacetic acid [PAA]) was described in a 1987 study by Bouvet and Grimont as part of a biotyping scheme of clinical Acinetobacter isolates (45, 119). Since then, the bacterial PAA catabolism pathway encoded by *paa* genes was defined in E. coli and Pseudomonas putida (120). A. baumannii *paa* genes have been shown to be essential for virulence in murine models of septicemia, catheter-associated urinary tract infections, and in a zebrafish infection model (121–123). *paa* genes were also essential for colonization and persistence by INseq experiments in the G. mellonella model but not in murine lung infection (27, 28). Transcription of the A. baumannii *paa* genes is affected by numerous regulators and environmental conditions, and they are often the most differentially expressed genes. For example, *paa* genes are upregulated in the presence of mucin, indole-3-acetic acid, and the combination of trimethoprim and sulfamethoxazole (123–125). By contrast, *paa* genes are downregulated with exposure to tigecycline or in the absence of regulators GacS and MumR (72, 121, 126). PAA catabolism also appears to directly modulate the host immune response. In a zebrafish model of infection, A. baumannii mutants lacking *paa* genes had decreased virulence that was due, at least in part, to excreted PAA serving as a neutrophil chemoattractant (122). Recently, PAA catabolism has been shown to contribute to resistance to antibiotics and oxidative stress (72, 123). Together, these studies establish that organic acids are important carbon sources for many Acinetobacter strains and that catabolism of PAA is critical for A. baumannii infection, immune evasion, and resistance to antimicrobials.

## AMINO ACIDS AS CARBON AND NITROGEN SOURCES AND LINKS TO VIRULENCE REGULATION

Proteinogenic α-amino acids can be used directly for protein synthesis or can serve as the sole carbon and/or nitrogen source for Acinetobacter spp. Arginine, aspartate, glutamate, and histidine can support growth of most Acinetobacter spp. *in vitro* when they are provided as sole carbon sources (1, 36). In addition to l-amino acids, A. baylyi can also use d-amino acids as carbon and nitrogen sources, including d-aspartate and d-asparagine (127). Mutants in genes involved in amino acid synthesis and transport genes were one of the primary groups selected against in a genome-wide INseq study to identify A. baumannii virulence factors in a murine model of lung infection, suggesting that amino acids are limited in the host environment (27).

Histidine catabolism has been shown to be important in A. baumannii infection. A. baumannii catabolizes histidine through the Hut system, with the four core enzymes HutHUIG and transporter HutT, that converts histidine to glutamate (Fig. 4) (128). The Hut system is conserved in pathogenic Acinetobacter spp. and HutH is required for utilization of histidine as a nitrogen source *in vitro* (129). A *hutH* mutant strain was severely attenuated in the lungs of a murine model of pneumonia, suggesting that histidine serves as a crucial nitrogen source during infection (129). The Hut system has also been implicated in nutrient zinc homeostasis and biofilm formation (67, 130). HutH binds zinc and is important for growth under low-zinc conditions (67). The authors posit that histidine-zinc complexes serve as a zinc reservoir in A. baumannii and that HutH-mediated histidine catabolism liberates free zinc (67). The multiple roles of HutH show the integration of metabolic strategies that A. baumannii has evolved to survive in the host. Histidine can also be degraded to histamine by histidine decarboxylase; however, histamine could not support Acinetobacter species growth, suggesting histamine synthesis is important for other processes such as acinetobactin synthesis and interaction with immune cells via the histamine receptor (36, 79, 131–133). Many amino acids, including histidine, glutamate, lysine, arginine, alanine, branched-chain amino acids, and ornithine, were detected in the extracellular lumen of the murine lung, suggesting that they could serve as potential pathogen nutrient sources during infection (129).

The change of the nutrient landscape during infection may be a cue to A. baumannii to change its transcriptional program to persist and adapt to a hostile host environment. Consistent with this paradigm, multiple transcriptional regulators have been identified that link Acinetobacter amino acid metabolism and virulence. For example, A. baumannii cysteine regulators Cbl and GigC have been shown to be important for virulence in a G. mellonella model of infection (134, 135). In E. coli, cysteine biosynthesis and sulfur acquisition are regulated by the LysR family transcriptional regulators CysB and Cbl (136). A. baumannii Cbl was recently reported to also activate expression of cysteine biosynthetic genes and other sulfur assimilation genes and is critical for G. mellonella infection (135). However, A. baumannii strains do not encode CysB. Instead, the LysR family transcription regulator GigC was identified as important in a genome-wide INseq screen of G. mellonella infection (28). A mutant strain lacking GigC has impaired growth in the absence of the cysteine, which is essential for virulence in murine lung and intraperitoneal infection models (134). Together, these findings suggest that GigC and Cbl coordinate expression of cysteine biosynthesis and sulfur acquisition in A. baumannii, which is critical for virulence. AvnR, a CheY-like response regulator, regulates virulence phenotypes, including adherence to A549 cells and pathogenicity in the G. mellonella model, and is required for using amino acids as nitrogen sources (137). A. baumannii *csrA* is required for growth in amino acid media, resistance to desiccation, and growth in human urine (18, 138, 139). A. baumannii CsrA is a homolog of the E. coli carbon storage regulator CsrA that regulates E. coli sugar catabolism; however, A. baumannii CsrA does not appear to be involved in sugar metabolism (138). Regulators important for A. baumannii infection are still being studied, and there are likely additional regulators linking virulence and amino acid metabolism.

Host proteins and metabolites are potential sources of nutrients and cues for changes in bacterial transcriptional programming during infection. Mucin is a glycoprotein secreted by lung epithelial cells during respiratory infections that can serve as the sole carbon and nitrogen source for A. baumannii growth *in vitro* and results in upregulation of PAA catabolism genes (124). The same study mentioned above reported that human pleural fluid changed the metabolism of phenylalanine in A. baumannii and resulted in enhanced immune evasion of human neutrophils (118). Therefore, host proteins and metabolites can alter A. baumannii gene expression, affecting metabolism and immune evasion.

Several studies have identified links between antibiotic effects and amino acid metabolism in A. baumannii. For example, polymyxin-resistant A. baumannii isolates induce perturbations of amino acid metabolism (140). To fight MDR or XDR A. baumannii, combination therapies such as colistin-doripenem, colistin-sulbactam, and polymyxin B-rifampin have been widely studied (141). These studies suggest that critical pathways such as lipid A, carbohydrate, nucleotide, energy, and amino acid metabolism are more effectively disrupted by combination therapies than monotherapies (142–144). Another study reported that amino acid transport may be involved in drug resistance. Disruption of the *carO* gene, which encodes an outer member protein, results in carbapenem resistance; CarO also has been shown to be required for l-ornithine uptake and is implicated in l-histidine uptake (130, 145). A recent study showed that some A. baumannii amino acids metabolism genes are downregulated upon interaction with THP-1 macrophages and in response to polymyxin B treatment (146), suggesting that amino acid metabolism may be compromised by host-imposed stresses and antimicrobials. Finally, l-lysine has been found to potentiate aminoglycosides against A. baumannii and other Gram-negative pathogens (147). Together these findings indicate that interfering with A. baumannii amino acid metabolism may synergize with host immunity and antibiotic therapies to fight infections with A. baumannii.

## OTHER AMINE COMPOUNDS AND THE POTENTIAL FOR IMMUNOMODULATION

In addition to using amino acids directly as carbon and nitrogen sources, some Acinetobacter spp. can utilize amino acid-derived metabolites and nonproteinogenic amino acids present in host tissues. Many Acinetobacter spp. can use β-alanine, 4-aminobutyrate/γ-aminobutyric acid (GABA), putrescine, and other amine compounds as carbon sources (1, 36). Carnitine is a quaternary amine that is synthesized from lysine in humans. A. baumannii growth on carnitine as the sole carbon and energy source requires the carnitine transporter Aci01347 (148). GABA is a neurotransmitter synthesized from glutamate that can serve as a carbon source for most Acinetobacter spp. (1). GABA catabolic genes are also transcriptionally regulated by the manganese-responsive MumR regulator, and a mutant lacking *gabT* is more susceptible to hydrogen peroxide stress (72). Urea is formed in humans as part of the urea cycle and is important in nitrogen excretion. As mentioned above, MumR also regulates expression of genes encoding urea metabolism enzymes, including the urea carboxylase MumC, which is important for utilizing urea as a nitrogen source (71). Recently, A. baumannii was reported to utilize kynurenine, a metabolite of l-tryptophan degradation, as the sole carbon source (45). Interestingly, the kynurenine cluster is almost exclusively present in the pathogens of the Acinetobacter calcoaceticus/A. baumannii (ACB) clade, and it is not present in nonpathogenic Acinetobacter spp. (45). Host cells degrade tryptophan via the kynurenine pathway, and its metabolites are known to control immune system homeostasis (149, 150). In P. aeruginosa, the level of kynurenine intermediate 3-OH-anthranilate was increased in bronchial alveolar lavage fluid at 12 and 24 h postinfection and depended upon an intact P. aeruginosa kynurenine pathway (151). Kynurenine production by P. aeruginosa has also been shown to promote its survival in the presence of human neutrophils by inhibiting reactive oxygen species production (152). Whether A. baumannii kynurenine catabolism affects virulence and the immune response has yet to be determined. Overall, pathogenic Acinetobacter spp. appear to have evolved strategies to utilize host amine compounds as carbon and nitrogen sources, and future work may investigate whether these pathways affect immune cell function and modulate the host-pathogen interaction.

## CONCLUSIONS AND FUTURE DIRECTIONS

In conclusion, recent work has identified numerous metabolic strategies Acinetobacter strains use to acquire nutrients in the host during infection. However, many open questions remain as to Acinetobacter nutrient acquisition and metabolism during infection. Pathogenic Acinetobacter spp. can degrade numerous amino acids and other amines as carbon sources, but their roles as nitrogen sources are less characterized. Similarly, sulfur acquisition is largely unexplored. Sulfate assimilation was found to be critical for persistence in a G. mellonella model of infection (28). It is unknown whether Acinetobacter spp. encode additional mechanisms to liberate host-specific sulfur sources, as described for other bacterial pathogens (153). Similarly, future research could address whether and how pathogenic Acinetobacter spp. acquire cofactors, amino acids, nucleotides, and additional nutrient metals from specific host niches.

Many of the mechanistic studies described here were conducted using A. baumannii clinical isolates from the 1950s, which replicate extracellularly during infection. Recent studies have shown that some modern A. baumannii clinical isolates are capable of replicating intracellularly in macrophages or epithelial cells (95, 154). Nutrients are often limited inside pathogen-containing vacuoles, and how A. baumannii acquires nutrients to sustain its metabolic demands is unknown. Additionally, many studies suggest that A. baumannii asymptomatic colonization of multiple body sites is associated with infection risk (8–10, 14–16, 146). However, we know very little about the strategies pathogenic Acinetobacter spp. use to survive as a commensal, including nutrient utilization. In a mouse model, thioredoxin-mediated reduction of secretory IgA promoted A. baumannii gut colonization (155). As mentioned above, one study approached this question by coculturing A. baumannii with upper respiratory tract microbiota species and found that A. baumannii inhibits commensal bacteria with acinetobactin (82). Another study investigated Acinetobacter calcoaceticus features that allow it to thrive in the gastrointestinal tract and identified relevant acid resistance, salt resistance, and nutritional flexibility (39). Abundance of Acinetobacter bacteria in the gut was positively correlated with meat consumption in adults with nonalcoholic hepatic steatosis (156), suggesting that diet could be important for gut colonization in certain populations. Finally, how A. baumannii regulates carbon source utilization by carbon catabolite repression in the complex milieu of nutrients in the host is unknown and likely shapes infection outcomes.

A. baumannii iron acquisition has been successfully targeted by the new antibiotic cefiderocol, illustrating the potential for essential nutrient acquisition pathways to be targeted for new therapeutics. However, cefiderocol resistance has already been identified in carbapenem-resistant A. baumannii, and therefore new therapeutics must be developed. Multiple studies have implicated the pentose phosphate pathway in the response to polymixin antibiotics that target the outer membrane (140, 142, 157), suggesting complex integration of central metabolism and antibiotic tolerance and resistance. Strategies to target Acinetobacter metabolism may be able to successfully synergize with existing antimicrobials and the host immune system. Targeting nutrient acquisition or metabolism would likely be effective against both hospital-acquired and community-acquired strains, a key benefit to this strategy. A better understanding of how nutrients modulate the host-pathogen interface during Acinetobacter infection may uncover new avenues to solve the problem of antimicrobial resistance.
